# Tuning the Properties of a UV-Polymerized, Cross-Linked Solid Polymer Electrolyte for Lithium Batteries

**DOI:** 10.3390/polym12030595

**Published:** 2020-03-05

**Authors:** Preston Sutton, Martino Airoldi, Luca Porcarelli, Jorge L. Olmedo-Martínez, Clément Mugemana, Nico Bruns, David Mecerreyes, Ullrich Steiner, Ilja Gunkel

**Affiliations:** 1 Adolphe Merkle Institute, University of Fribourg, Chemin des Verdiers 4, 1700 Fribourg, Switzerland; martino.airoldi@unifr.ch (M.A.); clement.mugemana@list.lu (C.M.); nico.bruns@unifr.ch (N.B.); ullrich.steiner@unifr.ch (U.S.); 2 POLYMAT, University of the Basque Country UPV/EHU, Avda. Tolosa 72, 20018 Donostia-San Sebastian, Spain; luca_porcarelli001@ehu.eus (L.P.); jolmedo001@ikasle.ehu.eus (J.L.O.-M.); david.mecerreyes@ehu.es (D.M.); 3 Institute for Frontier Materials, Deakin University, 221 Burwood Hwy, Burwood, VIC 3125, Australia; 4 Luxembourg Institute of Science and Technology, Materials Research and Technology Department, 5 rue Bommel-ZAE Robert Steichen, L-4940 Hautcharage, Luxembourg; 5 Department of Pure and Applied Chemistry, University of Strathclyde, Thomas Graham Building, 295 Cathedral Street, Glasgow G1 1XL, UK

**Keywords:** lithium batteries, solid polymer electrolytes, dual-ion and single-ion conductor, scalable cross-linked polymer, UV polymerization, tunable matrix

## Abstract

Lithium metal anodes have been pursued for decades as a way to significantly increase the energy density of lithium-ion batteries. However, safety risks caused by flammable liquid electrolytes and short circuits due to lithium dendrite formation during cell cycling have so far prevented the use of lithium metal in commercial batteries. Solid polymer electrolytes (SPEs) offer a potential solution if their mechanical properties and ionic conductivity can be simultaneously engineered. Here, we introduce a family of SPEs that are scalable and easy to prepare with a photopolymerization process, synthesized from amphiphilic acrylic polymer conetworks based on poly(ethylene glycol), 2-hydroxy-ethylacrylate, norbornyl acrylate, and either lithium bis (trifluoromethanesulfonyl) imide (LiTFSI) or a single-ion polymethacrylate as lithium-ion source. Several conetworks were synthesized and cycled, and their ionic conductivity, mechanical properties, and lithium transference number were characterized. A single-ion-conducting polymer electrolyte shows the best compromise between the different properties and extends the calendar life of the cell.

## 1. Introduction

Lithium batteries have an established position in energy storage for small and portable electronics. To grow into the larger transportation and grid storage roles, where they are forecast to be a disruptive force for the green economy, fundamental safety and energy density issues need to be resolved. Solid polymer electrolytes (SPEs) are a component with the potential to address these two issues, namely the flammability and the low capacity of current cell designs. Replacing the typical organic carbonate liquid electrolyte solvents with low-volatility solid polymers can increase capacity through compatibility with metallic lithium anodes, while limiting the associated risk of failure or fire by resisting lithium dendrite-related short circuits [1]. The principal factor preventing SPE uptake is their low ionic conductivity (<10^−4^ S cm^−1^), resulting from a transport mechanism that relies on polymer chain mobility which is compromised as modulus is increased [2,3]. 

Certain polymers like poly(ethylene glycol) (PEG), also denoted as poly(ethylene oxide) (PEO), have come close to providing the required conductivity and remain a common component in SPE research. Identifying good alternative polymer electrolytes to PEG, that are electrochemically stable, complex lithium ions and have low glass transition temperatures (*T*_g_) has proven challenging [4]. While PEG is found in numerous systems, its use as a homopolymer SPE is limited by its semi-crystalline nature, since crystalline regions are ionically less conducting than amorphous PEG regions, requiring heating above the melting point to achieve high conductivities [5,6]. However, at these elevated temperatures, PEG homopolymers lose the dimensional stability and the stiffness required to resist dendrite formation [1]. To address these challenges, multi-component polymer systems have emerged as a potential solution, attempting to decouple mechanical properties from ion conduction [7,8,9]. While PEG is still featured as the principal ion-conducting component in many of these systems, it is blended, cross-linked, and made into block copolymers (BCPs) in an attempt to preserve the Li-ion transport properties while simultaneously tailoring the mechanical properties of the SPE to resist dendrites.

The number of candidate systems is incredibly large and cannot be covered in detail here [4,7,9]. The focus of this article is on a PEG cross-linked 2-hydroxyethyl acrylate electrolyte (PHEA-*l*-PEG), where mechanical properties come primarily from the PEG crosslinker and from the intrinsic mechanical properties of the insulating HEA phase, stiffening the SPE while maintaining a disordered structure across a range of compositions [10]. This is in contrast to BCP systems, another typical class of SPEs, in which the microphase-separated morphology and its arrangement in the SPE depends very sensitively on the BCP composition, often significantly affecting ion-conductivity across the material [2]. Additionally, certain UV-cross-linked networks, such as the one explored here, are typically easier to synthesize than BCPs, and their disordered morphology should be insensitive to the kinetic limitations of nanostructures and to the surface effects, which plague BCP systems [11,12,13]. The challenge in cross-linked networks, as with any polymer electrolyte where Li-ion transport is linked to chain mobility, is finding the optimal balance between high stiffness, often accompanied by high *T*_g_ values, and low ion conductivity at high *T*_g_ [14]. This is particularly relevant when high-*T*_g_ single-ion conductors (SICs) are incorporated into SPEs to reduce electrode polarization associated with traditional dual-ion salts such as lithium bis (trifluoromethanesulfonyl) imide (LiTFSI). 

Single-ion-conducting polymer electrolytes, where the anion is tethered to the polymer backbone, are already a well-established class of electrolytes, with a large variety of compositions allowing various properties to be tailored [15]. The benefits of a SIC-doped electrolyte over a typical lithium salt-doped electrolyte (e.g., LiTFSI) reside in the increased Li transference number (*T*_#_), defined as the mobility of the Li-ion compared to that of its associated anion; for an ideal SIC, *T***_#_** = 1. Common lithium salts have reported transference numbers ranging from 0.5 to as low as 0.1, with typical values well below 0.5 [9,16]. These mobile anions result in charge concentrations near the electrodes, which translate to higher electrode polarization, a higher internal resistance, and have been linked to dendrite growth [17]. Based on the Chazalviel model, anion depletion at the negative electrode during charging leads to high electric field strengths, which in turn leads to an increase in electrodeposition and growth of Li dendrites. Previous work employing SICs has shown *T*_#_ values greater than 0.9 in a copolymer, reducing electrode polarization and exhibiting conductivity values of 10^−4^ S cm^−1^, resulting in good cycling performance in cells [18]. 

The conductivity mechanism of polymeric SICs is similar to general SPEs, with the glass transition temperature, the ion mobility, and charge delocalization contributing to overall effectiveness of lithium transport [15]. Since the underlying conductivity in both dual-ion and single-ion polymeric systems is usually linked to chain mobility, the strategy for optimizing SICs has similar issues to other SPEs. Increasing the mechanical stiffness and film forming properties, which resist lithium dendrite growth, will inevitably lower chain mobility and therefore the ion conductivity [12]. To decouple the antagonistic relationship between the stiffness and the ion conductivity in SICs, multi-component polymers, such as BCPs, have already been employed, where one phase of the system controls the mechanical properties of the electrolyte, while another provides ion conductivity [15,16,18]. Meek et al. have exploited a BCP-SIC to produce a highly conducting sample, but their work was limited primarily to polycation systems, which are not appropriate for lithium batteries [19]. Inceoglu et al. and Rojas et al. have synthesized BCP-SICs with ion conductivities >10^−4^ S cm^−1^ at 90 °C, but mechanical properties were not reported [16,20]. Both previous systems show increased conductivities as the BCP system disorders, freeing the lithium ions from the SIC phase and releasing them into the PEG. While the mechanical consequences of heating and doping are unclear, typically this leads to a significant reduction in mechanical properties [21]. While the SPE reported here is also a copolymer system, it is important to emphasize that it is not a BCP, thus avoiding ordered microphases, which can result in low ion conductivities [16,20]. Instead, crosslinking disrupts the conductivity-limiting crystallinity in PEG and imparts good mechanical properties without the need for a microstructure, an approach which was successfully exploited by Walker et al. and Zheng et al., demonstrating SPEs with conductivities >10^−4^ S cm^−1^ at 30 °C. Both of these systems employed the LiTFSI salt, but no cycling data are reported to demonstrate dendrite resistance. Applying SICs to these types of SPEs could be promising to maintain the high ion conductivities while extending cycling through reduced electrode polarization and dendrite growth [22,23]. 

The principal goal of this electrolyte study is therefore the development of simple SPE networks, which are tunable in terms of both their molecular design (different monomers) and their ion source (dual vs. SIC), to study fundamental relationships between molecular mobility, ion conductivity, and mechanical strength in disordered SPEs, i.e., in the absence of detrimental morphological order. To those ends, PHEA-*l*-PEG electrolytes doped with LiTFSI and a SIC were compared. Additionally, low-*T*_g_ HEA was substituted with the higher-*T*_g_ isobornyl acrylate (IBA) monomer. While several previously researched systems have similar components, this study focuses on one network (PHEA-*l*-PEG) and systematically explores the effect of component variation on the interplay of ion conductivity and mechanical modulus, and their effect on galvanostatic cycling.

## 2. Materials and Methods 

### 2.1. Materials

Lithium bis(trifluoromethanesulfonyl) imide (LiTFSI), 2-methyl-4’-(methylthio)-2-morpholinopropiophenone (Irgacure 907), 2-hydroxyethylacrylate, 96% (HEA), isobornyl acrylate (IBA) and reagent-grade methyl ethyl ketone (MEK) were purchased from Sigma-Aldrich Corp (Saint-Louis, MO, USA). Polyethylene glycol dimethacrylate (4 kg mol^−1^) (MA-PEG-MA) was purchased from Monomer-Polymer & Dajac Labs, Inc (Ambler, PA, USA). LiTFSI was vacuum dried overnight at 70 °C before use. The HEA and IBA monomers were purified by passing through a basic alumina column and stored refrigerated in opaque containers.

Poly[lithium 1-[3-(methacryloyloxy) propylsulfonyl]-1-(trifluoromethylsulfonyl) imide] (PLiMTFSI), with 3 degrees of polymerization (DP) equal to 14, 29, and 43 (DP14, DP29, DP43, respectively) were synthesized by RAFT polymerization in a 3 step procedure fully described elsewhere [24]. 

### 2.2. Methods

HEA and/or IBA monomers plus the MA-PEG-MA linker, neat, with LiTFSI, or with PLiMTFSI, were mixed in one pot. LiTFSI and PLiMTFSI were weighed in an Ar atmosphere glovebox (MBRAUN, Garching, Germany; <0.01 ppm O_2_ and H_2_O) before transfer to an ambient hood, where they were immediately mixed with MEK, MA-PEG-MA (4 kg mol^−1^), the relevant monomers (different *v/v* ratios of HEA/IBA), and Irgacure 907: PEG/(HEA/IBA) m/v 1/1, MEK and Irgacure 907 approx. 38 and 2 wt% respectively, without Li-ion source (LiTFSI or PLiMTFSI). A typical sample consisted of 200 mg PEG, 200 µl total monomer (HEA and IBA), 8 mg Irgacure 907, 300 µl MEK, Li-ion source (104 mg LiTFSI or 123 mg PLiMTFSI for *r* = 0.08). Lithium-containing samples were mixed to achieve a specific lithium ion to PEG ether oxygen ratio defined by *r* = [Li^+^]/[EO]. Solutions were capped and mixed with a magnetic stirrer at 50 °C until dissolved (typically under 20 min). Once dissolved, solutions were cast into PTFE dishes and exposed to UV (Dymax 5000-EC Flood Lamp (Dymax Corp, Torrington, CT, USA): λ = 320–390 nm, 225 mW cm^−2^, 400 W at 10.5 cm) for 1 min. The samples were then vacuum dried without a rinsing step in a Binder vacuum oven (BINDER GmbH, Tuttlingen, Germany) at 70 °C for approx. 24 h before transfer back into the glovebox for storage and characterization.

Attenuated total reflectance Fourier-transform infrared spectroscopy (ATR-FTIR): Spectra were recorded on a Perkin Elmer Spectrum 65 spectrometer between 4000 and 600 cm^–1^ with a resolution of 4 cm^–1^, averaging 7 scans per sample. SPE samples were dried in a Binder vacuum oven (BINDER GmbH, Tuttlingen, Germany) at 70 °C for approx. 24 h before characterization while precursor materials were measured without any treatment.

Cell assembly: Swagelok cells (Swagelok Switzerland, Wohlen, Switzerland) and stainless steel (ss) current collectors were used for electrochemical impedance spectroscopy (EIS), galvanostatic cycling (GC) and cyclic voltammetry (CV) measurements. All samples were assembled in an Ar glovebox (< 0.01 ppm O_2_ and H_2_O) with a spring to maintain cell pressure. Any sample/cell subjected to electrochemical testing was vacuum dried at 70 °C for a minimum of 24 h. GC testing was performed in symmetrical Li//Li cells, while CV testing employed Li//Cu cells for cathodic scans and Li//ss cells for anodic scans.

Dynamic mechanical analysis (DMA): DMA was performed with a dynamic mechanical analyzer (TA Instruments, New Castle, DE, USA) in tensile mode. The tests were conducted at a frequency of 1 Hz and a strain amplitude of 15 μm in the temperature range of 0 to 95 °C at a heating rate of 5 °C min^−1^. Rectangular films with a length of approx. 15 mm, a width of 5.60 mm and thicknesses between 0.10 and 0.50 mm were used.

Differential scanning calorimetry (DSC): DSC measurements were performed under N_2_ using a Mettler-Toledo STAR system (Mettler-Toledo, Greifensee, Switzerland) operating at a heating/cooling rate of 10 °C min^−1^.

Electrochemical chemical characterization: Swagelok cells were assembled using appropriate electrode pairs. EIS, GC, CV, and electrode polarization data were taken using a BioLogic SP-300 potentiostat (BioLogic, Seyssinet-Pariset, France). Swagelok cell temperature was controlled in a Binder oven with approx. a 1 h dwell time for temperature equilibration. The samples were tested in typical through-plane configurations. EIS: Typical frequency range 5 MHz to 1 Hz with 10 mV perturbation. The measured impedance was converted to a conductivity, σ = *L*/*RA*, where *R* is the resistance determined from equivalent circuit fitting, *L* is the thickness of the electrolyte layer, and *A* is the surface area contact of the electrode-electrolyte interface. GC was performed at 70 °C with a current density of 0.02 mA cm^−2^, 1 h positive current followed by 1 h negative current. CV was performed at 70 °C with a scan rate of 0.2 mV s^−1^.

Small-angle X-ray scattering (SAXS): SAXS was performed using a Rigaku NanoMAX-IQ SAXS camera (Rigaku Europe SE, Neu-Isenburg, Germany) equipped with a Cu target sealed tube source (MicroMax003 microfocus from Rigaku). Scattering data were collected with a Pilatus 100k detector (Dectris). The sample-to-detector distance was calibrated with a silver behenate standard.

Thermogravimetric analysis (TGA): TGA was performed with a Mettler Toledo thermogravimetric analyzer system (Mettler-Toledo, Greifensee, Switzerland) with a 10 °C min^−1^ heating ramp.

Limiting current fraction (*t*_#_): *t*_#_ was calculated from the steady-state current (*I*_ss_) divided by the initial current (*I*_0_) of a 10 mV electrode polarization test, in a symmetrical Li/SPE/Li cell at 70 °C. EIS was performed before and after polarization. *I*_0_ was calculated using Ohm’s law and *R*_0_ was determined from the initial EIS measurement. The Bruce–Vincent method was used to get an indication of the Li-ion mobility via the transference number (*T*_#_) [25]. Note that this approach is applicable to dilute electrolytes and its suitability for the non-dilute SPEs tested here is unclear.

## 3. Results

**Scheme 1:**
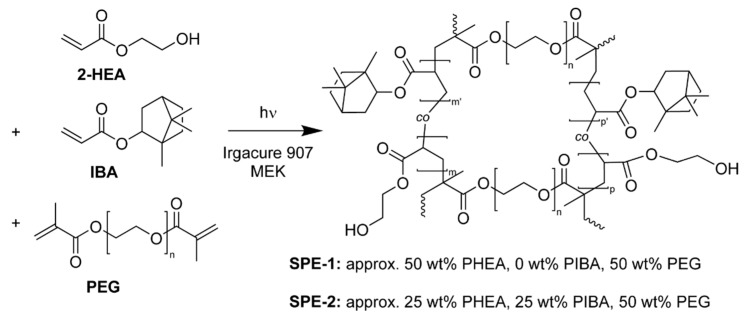
(SPE-1 and SPE-2) Synthetic route for UV-polymerized, poly(ethylene glycol) (PEG) cross-linked 2-HEA (PHEA-*l*-PEG) and PEG cross-linked 2-HEA-*co*-IBA ((PHEA-*co*-PIBA)-*l*-PEG) solid polymer electrolytes. Note that the lithium bis (trifluoromethanesulfonyl) imide (LiTFSI) salt is not depicted for clarity. The weight fractions of methyl ethyl ketone (MEK) and Irgacure 907 are approx. 38 and 2 wt% respectively, relative to the neat sample pre 1 min UV cure and overnight vacuum drying at 70 °C.

The SPE system investigated here is based on UV-curable, scalable polymer conetworks, which were developed as amphiphilic polymer conetworks by Bruns et al. for non-battery applications [26]. This system was selected to create disordered, continuous ion-conducting materials with high PEG volume fractions, which does not suffer from morphological conductivity penalties typical of nanostructured BCP electrolytes [2]. The initial cross-linked system (SPE-1) consists of LiTFSI salt-doped poly(2-hydroxyethyl acrylate) linked by poly(ethylene glycol) crosslinkers to form PHEA-*l*-PEG freestanding films; see Scheme 1. 

The HEA monomer was polymerized with a PEG linker of fixed molecular weight *M* = 4 kg mol^−1^, which is easily adjustable. The single-pot polymerization was initiated by UV exposure under ambient conditions, highlighting the simplicity and scalability of the process. Additionally, the synthesis protocol allows facile HEA monomer substitution to adjust the mechanical properties of the network. The platform also readily accepts dual and single ion Li sources.

To demonstrate the facility of monomer substitution as a route to engineer the mechanical properties of the electrolyte, a fraction of 2-hydroxyethyl acrylate was replaced by isobornyl acrylate (SPE-2); see Scheme 1. The underlying assumption motivating this substitution is that HEA endows the network with its mechanical properties and does not contribute to Li-ion conduction. Under this assumption, replacing portions of HEA with a stiffer monomer might provide a simple way to increase the elastic modulus of the network while maintaining its ion conductivity. IBA was selected because of its acrylate end group, which should polymerize at a rate similar as HEA, thus minimizing structural differences in the SPE samples.

**Scheme 2:**
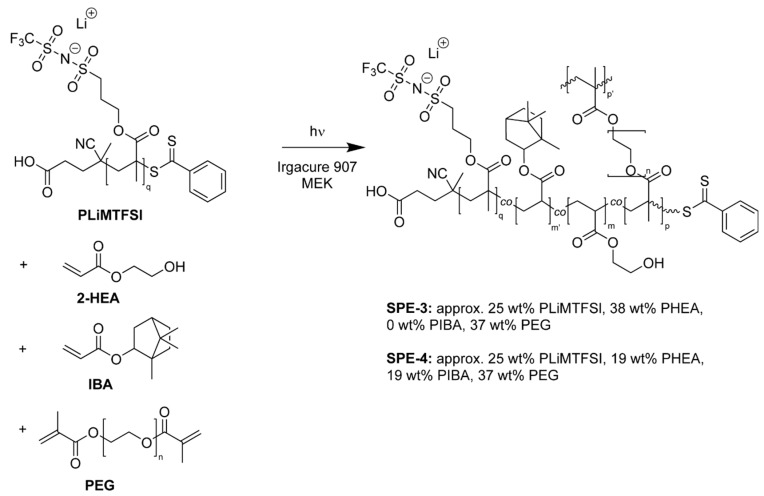
(SPE-3 and SPE-4) Synthetic route for UV-polymerized, PEG cross-linked 2-HEA-*co*-single ion conductor (PLiMTFSI) ((PHEA-*co*-PLiMTFSI)-*l*-PEG) and PEG cross-linked 2-HEA-*co*-single ion conductor (PLiMTFSI)-*co-*IBA ((PHEA-*co*-PIBA-*co*-PLiMTFSI)-*l*-PEG) solid polymer electrolytes. The weight fractions of MEK and Irgacure 907 are approx. 38 and 2 wt% respectively, relative to the neat sample pre 1 min UV cure and overnight vacuum drying at 70 °C.

In addition to the SPE-1 and SPE-2 systems, the SIC PLiMTFSI (full description available in previous work [24]) was linked to the PHEA-*l*-PEG network, resulting in SPE-3; see Scheme 2. 

PLiMTFSI was synthesized via reversible addition-fragmentation chain-transfer (RAFT) polymerization. Due to fact that only one end of the PLiMTFSI can be re-initiated in the UV-induced radical polymerization, it is incorporated as a dangling chain into the polymer networks, changing the network structure relative to SPE-1 and SPE-2. During polymerization, the SIC can also be considered a chain start for PHEA, followed by a PEG crosslinker, followed by another PHEA chain, forming (PHEA-*co*-PLiMTFSI)-*l*-PEG. The PLiMTFSI homopolymer, which is a SPE in its own right, has poor mechanical properties and its conductivity (<10^−7^ S cm^−1^ at 25 °C) lies well below the target values for a working cell (>10^−4^ S cm^−1^). The motivation for the integration of PLiMTFSI into the PHEA-*l*-PEG network is thus the improvement of the mechanical properties of the SIC SPE as well as an improvement in the ion transport [24]. 

As mentioned above, the immobile anion of the SIC contributes to dendrite suppression by reducing charge depletion at the Li electrode [10]. The addition of the SIC further permits SPE tailoring through changing the degree of polymerization of the PLiMTFSI. In this way, the Li-ion concentration can be held constant while the SIC dangling chain length is changed. This is yet another distinction between a polyelectrolyte and a traditional salt-doped electrolyte, where the salt concentration is linked to the mechanical properties in a more intimate way. In the case of LiTFSI, increasing the salt concentration increases the Li-ion concentration, thereby increasing the physical crosslinking of the Li ions between EO groups. This raises *T*_g_ and lowers the conductivity [6,14]. In PLiMTFSI containing samples the Li-ion concentration can be optimized through the molecular weight of the SIC, and then mechanical properties can be adjusted through its degree of polymerization. In addition, the mechanical properties of the SIC system can be modified through monomer substitution with IBA (SPE-4); see Scheme 2. 

For the SPEs studied here (SPE-1–SPE-4), monomer conversion and incorporation of the precursors into the respective network were confirmed based on ATR-FTIR (Figures S4–S8). Successful polymerization was additionally confirmed based on extraction experiments of neat SPE-1 (see Supplementary Materials).

**Figure 1:**
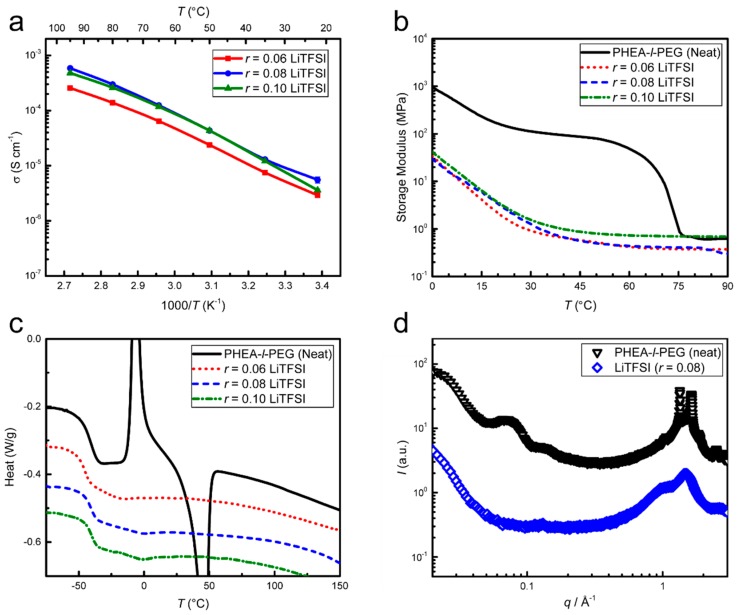
Characterization of SPE-1 as a function of LiTFSI salt concentration. (**a**) The conductivity measured by electrochemical impedance spectroscopy (EIS) shows increases with *r* and temperature, but plateaus at *r* = 0.08. (**b**) Dynamic mechanical analysis (DMA) shows a decrease in storage modulus (softening) upon salt addition. (**c**) Differential scanning calorimetry (DSC) (2nd heating at 10 °C min^−1^) shows that salt addition eliminates sample crystallinity, leaving *T*_g_ ≈ −40 °C unaffected. (**d**) X-ray scattering profiles exhibit sharp reflections at scattering vectors *q* = 1.2 Å^−1^–1.5 Å^−1^ in the neat sample, which are reduced to a broad signal for *r* = 0.08, indicating that salt addition eliminates crystallinity. The broad peaks in the 0.02 Å^−1^–0.2 Å^−1^ *q*-range indicate a disordered morphology on the nm-length scale, which disappears upon salt addition.

PHEA-*l*-PEG (SPE-1) with varying LiTFSI salt concentrations was studied initially, targeting conductivities >10^−4^ S cm^−1^ and maximal storage moduli at 70 °C. Samples with *r* (defined by [Li^+^]/[EO]) ≥ 0.08 showed the highest conductivity with *σ* > 2 × 10^−4^ S cm^−1^ and similar moduli of ≈1 MPa for all tested *r* values at 70 °C (Figure 1a,b).

Differences in conductivity for the various salt concentrations cannot be linked to the glass transition temperature, since *T*_g_ is unaffected by the *r*-value; see Figure 1c. The X-ray scattering data show a disordered structure and the elimination of crystallinity upon the addition of LiTFSI salt; see Figure 1d.

**Figure 2:**
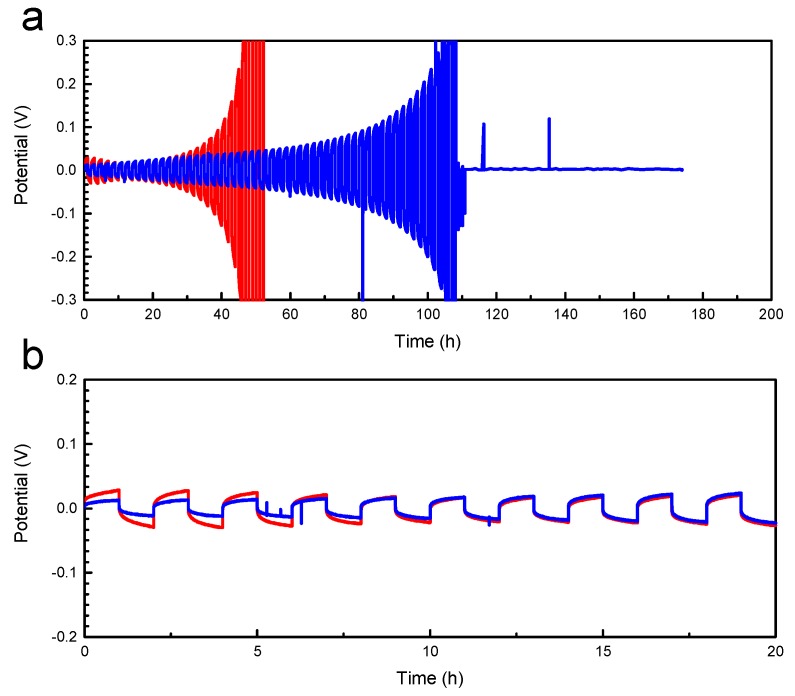
Galvanostatic cycling of SPE-1. (**a**) Data of the best (blue) and worst (red) performing cells show stable cycling up to 20 and 50 cycles (40 h and 100 h, respectively) before a significant overpotential develops. (**b**) Similar initial voltage response over one-hour half cycles. The tests were performed at 70 °C, with LiTFSI content corresponding to *r* = 0.08, in a symmetrical Li/SPE/Li cell with 1 h forward current followed by 1 h reverse current at 0.02 mA cm^−2^.

Galvanostatic cycling between symmetric metallic lithium electrodes in Figure 2 exhibits an initially low electrolyte resistance and decent cycling performance, which deteriorates steadily after 40 h (worst performer) to 100 h (best performer) before failure.

**Figure 3:**
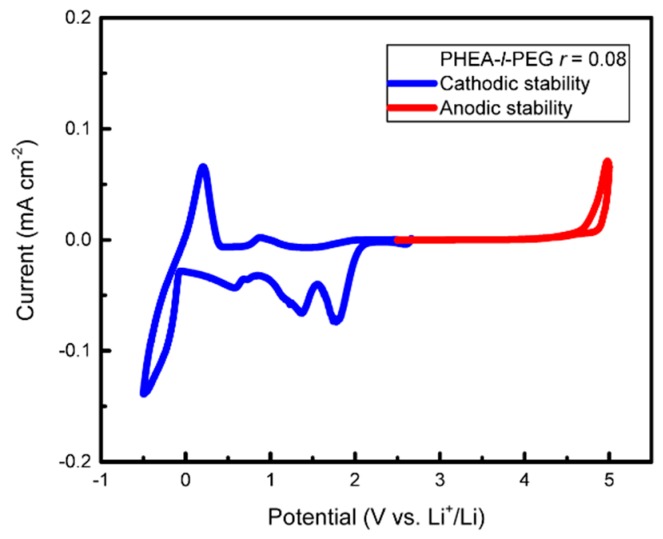
Cyclic voltammetry of SPE-1. The data show the plating and stripping of Li at approx. 0 V and stability up to 4.5 V. The peaks between 0.5 and 2 V show a mild cathodic instability of the system. The tests were performed at a scan rate of 0.2 mV s^−1^, 70 °C, *r* = 0.08, in a Li/SPE/stainless steel cell (anodic scan), and a Li/SPE/Cu cell (cathodic scan).

The cyclic voltammetry data of Figure 3 show a relatively large voltage stability window (0–4.5 V) overall for SPE-1. However, the peaks in the 0.5–2 V vs. Li^+^/Li range show the presence of reduction processes, indicating a limited cathodic stability in this voltage range.

**Figure 4:**
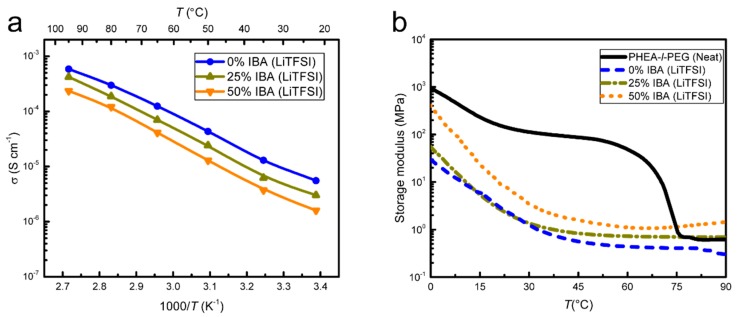
Comparison of SPE-2 with SPE-1 at an LiTFSI concentration of *r* = 0.08. SPE-2 with LiTFSI shows (**a**) an increase in conductivity as a function of temperature for all samples, as determined by EIS. Replacing 25% and 50% of HEA in SPE-1 by isobornyl acrylate (IBA) (resulting in SPE-2) shows a conductivity decrease with increasing IBA content. (**b**) DMA of SPE-2 shows an increase in the temperature-dependent storage modulus with increasing IBA content.

HEA monomer substitution with the IBA monomer, resulting in PHEA-*co*-IBA-*l*-PEG (SPE-2), was investigated to explore the possibility of modifying the mechanical properties of SPE-1. Adding the IBA monomer did increase the storage modulus relative to SPE-1 at similar LiTFSI concentrations, but there was a drop in ionic conductivity upon electrolyte stiffening (Figure 4a,b).

Despite the reduced conductivity upon the addition of IBA, fully replacing the HEA monomer with IBA fully eliminates the cathodic instability of the electrolyte in Figure 3, as shown in Figure S1.

**Figure 5:**
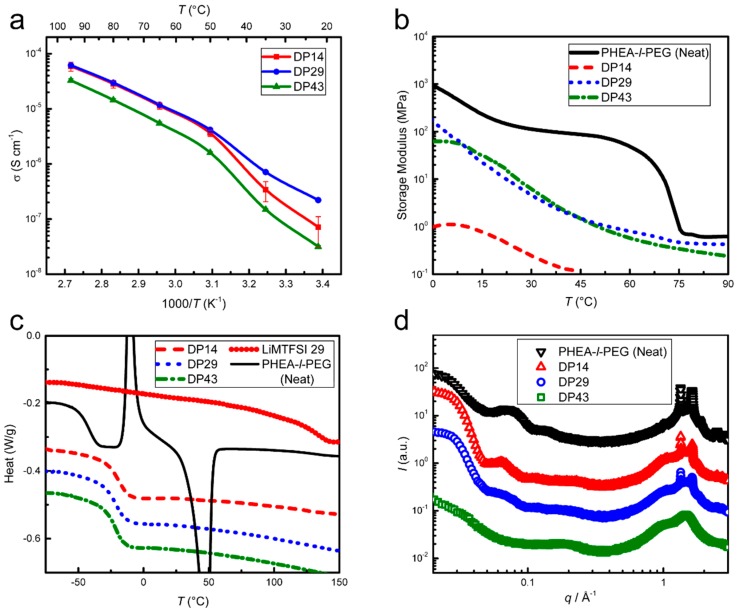
Characterization of SPE-3. Three different degrees of polymerization of the single-ion conductor (SIC) PLiMTFSI (DP14, DP29, DP43) for a constant Li-ion concentration (*r* = 0.08) are shown. (**a**) EIS shows an increase in conductivity with increasing temperature, with a shoulder between 35 and 50 °C. The conductivity is decreased with increasing DP (decreasing number of SIC chains), particularly below 50 °C. (**b**) DMA of SPE-3 shows a decrease in storage modulus (softening) with temperature and with a lower degree of polymerization, from DP43 and DP29 to DP14. (**c**) DSC (2nd heating at 10 °C min^−1^) shows a lack of crystallinity and similar *T*_g_ values of ≈−20 °C in all Li-ion-doped samples, which are higher than SPE-1 and lower than the PLiMTFSI homopolymer. (**d**) X-ray scattering shows reduction of crystallinity with increasing DP, with DP43 being completely amorphous. All samples lack well-defined reflections in the small-angle X-ray scattering (SAXS) *q*-range indicating the absence of morphological organization, implying a disordered arrangement of crystalline domains. The reduction in crystallinity is mirrored by a reduction of form factor scattering in the SAXS regime, indicating the deterioration in the structural arrangement of the (diminishing) crystalline domains.

To investigate strategies to increase cycle life, the ion source was modified from LiTFSI (dual-ion, SPE-1) to PLiMTFSI (single-ion, SPE-3), decreasing the overall conductivity from approx. 2 × 10^−4^ to 2 × 10^−5^ S cm^−1^ at 70 °C, while the storage modulus remained unchanged from SPE-1 (approx. 1 MPa at 70 °C); see Figure 5a,b.

**Figure 6:**
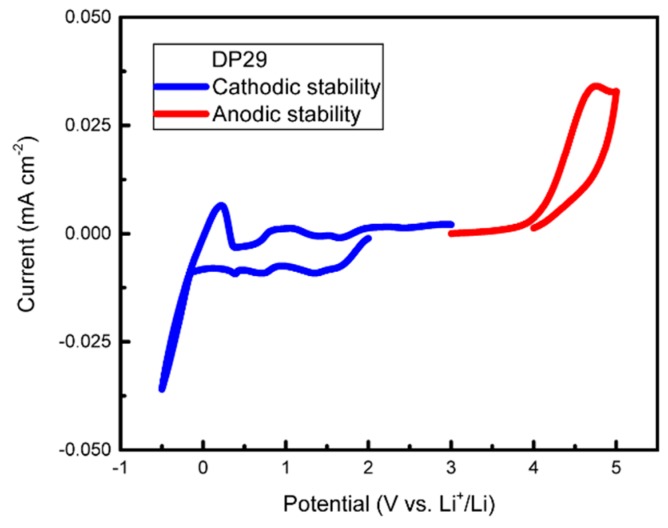
Cyclic voltammetry of SPE-3 DP29. Data showing plating and stripping of Li at 0 V and stability up to 4 V. The peaks between 0.5 and 2 V indicate a slight cathodic instability. CV cycling was performed at a scan rate of 0.2 mV s^−1^, 70 °C, *r* = 0.08, in a Li/SPE/stainless steel cell (anodic scan), and in a Li/SPE/Cu cell (cathodic scan).

The drop in conductivity relative to SPE-1 probably stems from an increase in *T*_g_ (Figure 5c), since there is no evidence for structure formation in the bulk (SAXS data in Figure 5d). The increase in *T*_g_ by approx. 20 °C upon the incorporation of the less mobile PLiMTFSI into the electrolyte agrees with the prediction of the Fox Equation for miscible blends [27]. The cyclic voltammetry results of SPE-3 are similar to SPE-1, with a slightly smaller stability window of 0–4 V; see Figure 6.

**Figure 7:**
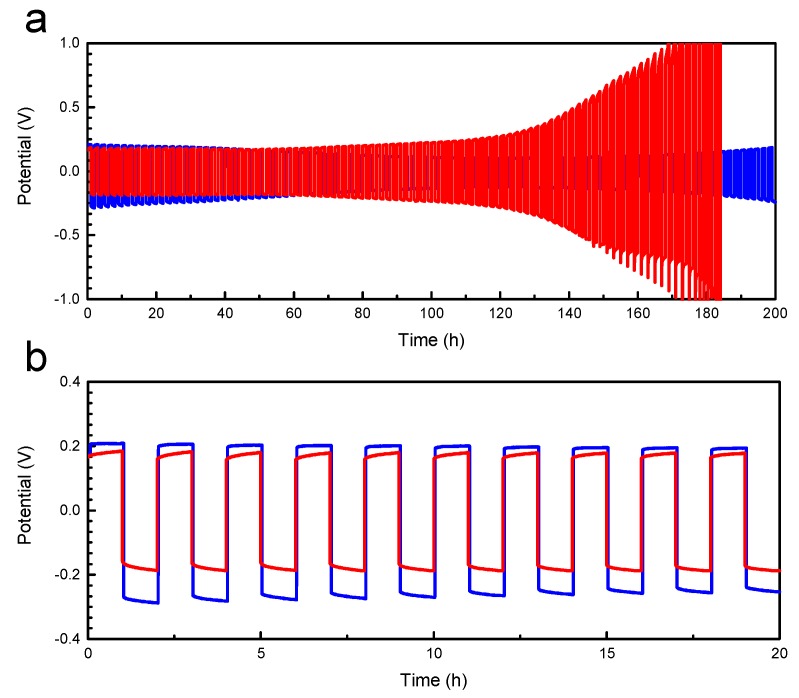
Galvanostatic cycling of SPE-3 DP29. (**a**) Data of a best (blue) and worst (red) performing cell show stable cycling up to 60 and >100 cycles (120 h and >200 h, respectively) before a significant overpotential develops. (**b**) Similar initial voltage response over the hour half cycles, with flat overpotentials. The tests were performed at 70 °C, and *r* = 0.08, in a symmetrical Li/SPE/Li cell with 1 h forward current followed by 1 h reverse current at 0.02 mA cm^−2^.

Interestingly, despite the decreased conductivity, and a similar cathodic stability compared to SPE-1, the galvanostatic cycling performance of SPE-3 was superior to SPE-1 (Figure 7).

**Figure 8:**
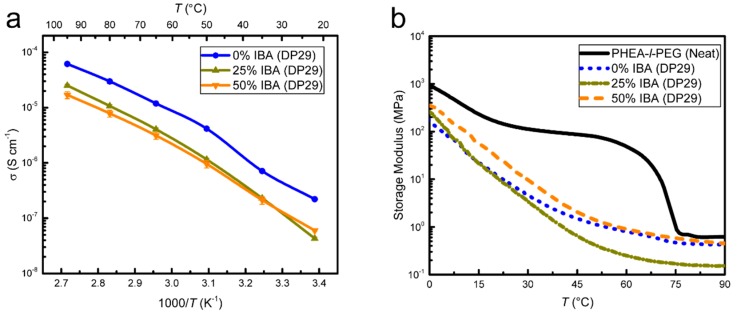
Comparison of SPE-4 with SPE-3 for DP29 and a Li-ion concentration of *r* = 0.08. (**a**) EIS shows a general increase in conductivity as a function of temperature for all samples, with a lower conductivity for IBA containing electrolytes. (**b**) DMA of SPE-4 shows a decrease in storage modulus (softening) as a function of temperature for all samples.

Substituting the HEA monomer by IBA (converting SPE-3 to SPE-4) was ineffective in stiffening the SPE but lowered overall conductivity (Figure 8a,b). In contrast to SPE-2 (Figure 4), where the addition of IBA more than doubled the storage modulus at 70 °C, the storage modulus of SPE-4 was reduced only for the sample with 25% IBA content and nearly identical for 0% and 50% IBA content.

All samples tested for thermal stability with TGA (SPE-1, SPE-3, SPE-4) showed no degradation below 200 °C (Figure S2).

## 4. Discussion

Based on conductivity alone, SPE-1 with *r* = 0.08 is the best performing electrolyte (Figure 1, Figure 4, Figure 5 and Figure 8), reaching a maximum conductivity of 6 × 10^−4^ S cm^−1^ at 95 °C. At these temperatures, the storage modulus of approx. 1 MPa is far below the 7 GPa required to resist dendrite growth according to the Monroe and Newman model [28]. This might suggest that SPE-1 is not an effective electrolyte for use with Li metal, but it should be noted that the 7 GPa minimum modulus value is based on a model that simply establishes a surface tension value for the deposition of Li metal. This model does not consider electrochemical or charging effects on Li nucleation. Stone et al. and Khurana et al. have shown effective dendrite resistant polymer electrolytes with elastic moduli at 0.1 GPa and 0.1 MPa, respectively, suggesting that the 1 MPa modulus of SPE-1 may be sufficient to prevent Li-dendrite growth during cycling [10,29]. 

Although all tested salt concentrations (*r* = 0.06–0.10) were effective in eliminating PEG crystallinity (see *r* = 0.08 in Figure 1c), maintaining decent conductivity below the PEG melting point (*T*_m, PEG_ ≈ 55 °C), this is only a minor point since the expected operating temperature of SPE-containing cells typically lies above the PEG melting temperature, where the conductivity is highest. The fairly constant value of *T*_g_ (approx. −40 °C) in all salted and unsalted samples (Figure 1c) indicate that the change in conductivity is most probably related to the effective ion concentration and ion mobility rather than salt dissociation and mechanical properties (Figure 1b). The salt dissociation across the Figure 1 samples should be similar, since it is influenced by the chemical environment of the SPE-1 matrix versus the LiTFSI lattice energy, which is constant. The optimal ion concentration of approx. *r* = 0.08 is in accordance with optimal values reported for similar PEG-based systems [12,30]. The drop in conductivity for SPE-1 *r* = 0.10 probably arises from an ion mobility decrease caused by ion pairing, rather than a polymer morphology change, since the X-ray scattering data show morphological disorder upon salt addition (Figure 1d).

Galvanostatic cycling (2 h cycle @ 0.02 mA cm^−2^) of two SPE-1 samples (*r* = 0.08 at 70 °C) showed excellent performance over 10 cycles (Figure 2), with a maximum generated potential of approx. 0.03 V, corresponding to a resistance of approx. 1.5 kΩ cm^−2^. As the cycle-life test continued, both samples saw rapid increases in potential, indicating cell failure possibly due to a combination of the formation of a high-resistance solid electrolyte interphase (SEI) and/or the formation of dendrites that short circuited the cell. The cyclic voltammetry data in Figure 3 show a weak cathodic instability below 2 V vs. Li^+^/Li, which may be indicative of continuous electrolyte reduction and the deposition of a resistive layer on one of the electrodes. Since PEG plus LiTFSI is known to be electrochemically stable between approx. 0 and 4 V vs. Li^+^/Li [2,9], it is likely that HEA is the unstable component. Therefore, substituting HEA for a stiffer, more electrochemically stable monomer, such as IBA (SPE-2), may not only improve the mechanical stability of the SPE against dendrite formation but also reduce HEA degradation, possibly extending the cycle life of the cell. Unfortunately, the stiffening effects of IBA (more than doubling the storage modulus at 70 °C) were offset by a reduction in conductivity by a factor of 5 (Figure 4). For the IBA composition, where a substantial storage modulus was realized (SPE-2 with 50% IBA (LiTFSI)), the conductivity was below the 10^−4^ S cm^−1^ target value at 70 °C. Even though IBA is not an ideal choice in the dual-ion system, CV results of SPE-2, in which all the HEA was replaced with IBA, did eliminate any cathodic instability of the electrolyte (Figure S1), demonstrating that HEA in SPE-1 was probably reducing. In the HEA/IBA containing networks, there is clearly a trade off between good conductivity (SPE-1) and electrochemical stability (SPE-2 100% IBA).

An alternative approach to reduce dendrite formation from metallic lithium anodes relies on replacing dual-ion conductors (LiTFSI) with single-ion conductors, such as PLiMTFSI. This may circumvent the requirement of high storage moduli SPEs, which may be too stiff to allow chain motion (reducing ion conductivity), or complicate cell construction (non-conformal electrolyte-electrode contact). Based on the so-called Chazalviel Model, anion depletion near a Li-metal electrode in dual-ion electrolytes causes a space charge that favors dendrite growth. Therefore, a SIC in a softer system might address the issue of dendrite-limited cycle life by attaching the anion to the electrolyte matrix, eliminating the need for a stiff electrolyte, which reduces the ion conductivity.

Based on SPE-1 results and work by Rojas et al. and Porcarelli et al., a lithium ion to ether oxygen ratio of *r* = 0.08 was selected for all SPE-3 samples [18,20]. The SPE-3 results show a clear effect of the PLiMTFSI chain length on conductivity, especially at lower temperatures, which does not seem to be correlated with a stiffening of the matrix (Figure 5a,b). Note that the Li-ion concentration is constant in all of these samples, implying that changes in the conductivity and mechanical properties arise from changes in the network structure related to the PLiMTFSI length. The intermediate degree of polymerization (DP29) maximizes the SPE-3 performance.

DMA results show reduced storage moduli for all three PLiMTFSI samples relative to the neat sample, with DP14 significantly softer than the other two. PLiMTFSI links to the network with only one chain end, forming “dangling chains”. This reduces the density of chemical cross-links in the network, thereby lowering the storage modulus of SPE-3 compared to SPE1. Note that the storage moduli of DP29 and DP43 are well above 0.1 MPa, the minimum value reported to resist dendrite formation by Khurana et al. [10]. 

The lack of an inverse relationship between storage modulus and ion conductivity is however surprising. Interestingly, DP14 matches the ion conductivity of DP29 at high temperatures, while falling below it at low temperatures, despite a lower storage modulus across the entire temperature range. The ion conductivity of DP43, on the other hand, is lower than that of DP29 by a factor of 5–10, despite a very similar storage modulus across the entire temperature range. Clearly, in SPE-3, it is not the overall network mobility (quantified by the storage modulus) alone that governs the ion conductivity. Since dangling chains typically are more mobile than fully coordinated network strands, it might well be that the mobility interplay of these two network components plays a role, potentially providing a different approach to tune the interplay of mechanical and electrochemical properties in ion-conducting polymeric networks.

A further interesting detail in Figure 5a is the conductivity shoulder in all samples between 40 and 50 °C. In PEG homopolymer SPEs, a similar variation in the conductivity is attributed to PEG melting. In SPE-3, however, DSC results show no measurable crystallinity in all samples (Figure 5c). For PLiMTFSI-containing networks, it is more likely that the binding energy of the Li to the PLiMTFSI is overcome in this temperature range.^18^ A further possible explanation involves the entire network and its ability to dissociate the Li ions. In work by Porcarelli et al., PEG is the only block competing to dissociate the lithium ion bound to the single-ion-conducting polymer. In contrast, our network contains HEA as well as PEG, providing a different electronic environment. Similar conductivity effects due to network backbone differences of SPEs have been reported by Doyle et al. [17]. 

The incorporation of PLiMTFSI into the network significantly reduces PEO crystallinity, an effect that increases with increasing PLiMTFSI molecular weight. Interestingly, wide-angle peaks corresponding to PEO crystallinity are discernible for DP14 and DP29 (Figure 5d) despite a lack of crystallization and melting signatures in the DSC curves in Figure 5c. Accompanied by the disappearance of the well-defined wide-angle peaks with increasing PLiMTFSI molecular weight is the reduction in the small-angle structure of the scattering profiles, indicating increasing structural disorder. This is in contrast to microphase-separated BCP SIC systems where low-temperature self-assembled morphologies prevent Li-ion diffusion from the SIC into the PEG domains [16]. 

Compared to the SPE-1 LiTFSI dual-ion system, the cycle life of SPE-3 in a symmetrical Li cell under the same test conditions is significantly longer (Figure 7). The electrolyte was able to reversibly transport Li ions from 60 to over 100 cycles (120 h and 200 h, respectively), before a rapid potential increase sets in. There is also a relatively uniform potential response over numerous cycles, in the range of +/-0.2 V vs. Li^+^/Li. The decreasing potential over the first 60 cycles of the best performer suggests the formation of a low resistance SEI layer, increasing the efficiency of lithium transport with time. The absence of voltage spikes and the continued cell operation in general show that dendrite-related short circuits across the electrolyte are avoided in the time frame of the experiment. Although SPE-3 shows higher resistance than SPE-1 (approx. 10 kΩ cm^−2^ vs. 1.5 kΩ cm^−2^), the increase in the amount of charge passed before failure at the same current confirms the benefits of a single-ion system. Note also that the same cathodic instability seen in SPE-1 is present in SPE-3 (Figure 6). While this can again be attributed to the reducing effects of HEA, the formed reduction products do not seem to have the similar negative effect as in SPE-1, suggesting that SPE-1 failure may ultimately be caused by dendrite growth. This demonstrates an interesting aspect of comparing a SIC vs. LiTFSI in the same network, demonstrating particularly the benefits of the lack of electrode polarization for the formation of reduction products that extend electrolyte calendar life.

The so-called “limiting current fraction” in SPE-1 and SPE-3 can be expressed as *t*_#_ = *I*_ss_/*I*_0_, with values of approx. 0.20 for SPE-1 and 0.85 for SPE-3. Upon correction for the kinetic resistances with the Bruce–Vincent Method, these values fall to approx. 0.50 and 0.70 for SPE-1 and SPE-3 respectively (Figure S3). These numbers are sometimes mistakenly referred to as transference numbers (*T*_#_), but as Doyle et al. and others have shown, there is a known difference between *T*_#_ and any transport number (e.g., *t*_#_) in non-dilute systems [4,31,32,33,34]. Here, we use *t*_#_ to qualitatively evaluate specific electrolytes, in the absence of the activity coefficient corrections required for non-ideal, concentrated SPEs. The limiting current fractions are reported to show their agreement with similar single and dual-ion systems in the literature [9,18,20]. 

The ion conductivities of IBA monomer substituted networks with the SIC electrolyte (SPE-4, Scheme 2) were similarly poor as those of SPE-2, but without the storage modulus improvements of SPE-2 (Figure 8). Again, the stiffer IBA may be reducing PEG chain motion, thereby reducing the Li-ion mobility, although a stiffening of the overall matrix of SPE-4 is not witnessed by the DMA results (Figure 8b). Beyond this simple argument, a complex process involving the electronic environment of the non-conducting portions of the system and their contribution to Li dissociation from the PLiMTFSI could be limiting the conductivity, but this is speculative. In SPE-4, adding IBA has only negative effects, lowering the conductivity while leaving the mechanical properties unchanged. While this does not disqualify monomer addition as a means to improve the overall storage modulus, it is clear that the relationship between mechanical and conductive properties is more complex than previously assumed. In both IBA-containing systems, SPE-2 and SPE-4, the ion conductivity is reduced relative to the HEA-only analogues, in spite of a low amount of network stiffening. Additionally, the cathodic instability in SPE-1 and SPE-3 does not seem to be relevant since the cycle life of SPE-3 does not seem to be strongly affected by it.

## 5. Conclusions

The principal aim of this project was to design a scalable, Li-ion-conducting polymer network, in which the ion conductivity and mechanical stiffness can be independently adjusted. This tunability allowed us to directly study important SPE metrics, such as the ion conductivity, glass transition temperature, storage modulus and cycle life, and their relationship to ion-source and monomer selection in a single system. Considering the four different classes of electrolytes explored (SPE-1–SPE-4), the single-ion-conducting SPE-3 offers the best combination of conductivity, mechanical properties, and cycle life, with room for further optimization in terms of Li-ion concentration and polymer architectures. The storage moduli of several of the tested electrolytes exceed the 0.1 MPa which may be adequate to resist Li dendrite growth, but a marked difference in the cycle life of the dual-ion (LiTFSI) vs. single-ion (PLiMTFSI) systems was found [10]. This finding illustrates the complicated interplay of ion transport and electrolyte stiffness to deliver a commercial solid polymer electrolyte for lithium batteries. This study also confirms that a single metric, e.g., ion conductivity, without transport numbers and cycle testing, is not sufficient to establish the effectiveness of a solid polymer electrolyte. The facile and scalable film production protocol described here provides a straightforward, single-pot synthesis that results in a relatively quick method for testing new monomers and ion sources. The simplicity and flexibility of this UV-curable electrolyte that can be processed under ambient conditions can serve as a robust platform to test specific monomer and salt properties to eventually develop a commercial polymer electrolyte.

## Supplementary Materials

The following are available online at https://www.mdpi.com/2073-4360/12/3/595/s1. Figure S1: Cyclic voltammetry of SPE-1 vs. SPE-2; Figure S2: TGA of 4 SPEs–SPE-1 (neat), SPE-1 (*r* = 0.08), SPE-3 (*r* = 0.08), SPE-4 (50% IBA, *r* = 0.08); Figure S3: Polarization and EIS for transference number of (a) SPE-1 and (b) SPE-3; Figure S4: ATR-FTIR of 2-HEA, LiTFSI, PEG, neat SPE-1 (*r* = 0), and LiTFSI-doped SPE-1 (*r* = 0.08); Figure S5: ATR-FTIR of 2-HEA, P (LiMTFSI), PEG, neat SPE-1 (*r* = 0), and SPE-3 containing P (LiMTFSI) (*r* = 0.08); Figure S6: ATR-FTIR of SPE-2 and SPE-4 compared to that of SPE-1 (0% IBA) at a constant Li-ion concentration of *r* = 0.08; Figure S7: ATR-FTIR of 2-HEA, PHEA, PEG, and neat SPE-1 (*r* = 0) in the hydroxyl group stretching region; Figure S8: ATR-FTIR of 2-HEA, PHEA, and neat SPE-1 (*r* = 0) in the stretching region of the carbon–oxygen bond of the 2-HEA monomer.
