# Supplementary material for: Tuning the Properties of a UV-Polymerized, Cross-Linked Solid Polymer Electrolyte for Lithium Batteries
Source: Polymers (Basel). 2020 Mar 5;12(3):595. doi: 10.3390/polym12030595 (PMC7182867; doi:10.3390/polym12030595)
Supplement: Supplementary file 1 [file polymers-12-00595-s001.pdf]

Supplementary Materials

Article

Tuning the Properties of a UV-Polymerized, Cross-Linked Solid Polymer Electrolyte for Lithium Batteries

Preston Sutton [1,*], Martino Airoldi [1], Luca Porcarelli [2,3], Jorge L. Olmedo-Martínez [2], Clément Mugemana [1,4], Nico Bruns [1,5], David Mecerreyes [2], Ullrich Steiner [1] and Ilja Gunkel [1,*]

1 Adolphe Merkle Institute, University of Fribourg, Chemin des Verdiers 4, 1700 Fribourg, Switzerland; martino.airoldi@unifr.ch (M.A.); clement.mugemana@list.lu (C.M.); nico.bruns@unifr.ch (N.B.); ullrich.steiner@unifr.ch (U.S.)
2 POLYMAT, University of the Basque Country UPV/EHU, Avda. Tolosa 72, 20018 Donostia-San Sebastian, Spain; luca_porcarelli001@ehu.eus (L.P.); jolmedo001@ikasle.ehu.eus (J.L.O.-M.); david.mecerreyes@ehu.es (D.M.)
3 Institute for Frontier Materials, Deakin University, 221 Burwood Hwy, Burwood, VIC 3125, Australia
4 Luxembourg Institute of Science and Technology, Materials Research and Technology Department, 5 rue Bommel-ZAE Robert Steichen, L-4940 Hautcharage, Luxembourg
5 Department of Pure and Applied Chemistry, University of Strathclyde, Thomas Graham Building, 295 Cathedral Street, Glasgow G1 1XL, UK
* Correspondence: preston.sutton@unifr.ch (P.S.); ilja.gunkel@unifr.ch (I.G.)

Electrochemical Stability

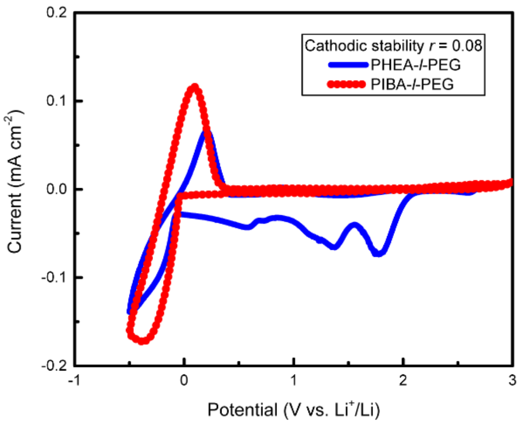

Figure S1: Cyclic voltammetry of SPE-1 vs. SPE-2. Data show plating and stripping of Li at 0 V for both samples, while the reduction peaks from 0.5-2 V of SPE-1 (100% HEA) are eliminated in SPE-2 (100% IBA). The tests were performed at a scan rate of 0.2 mV s^{-1}, 70 °C, r = 0.08, in a Li/SPE/Cu cell.

Thermal Stability

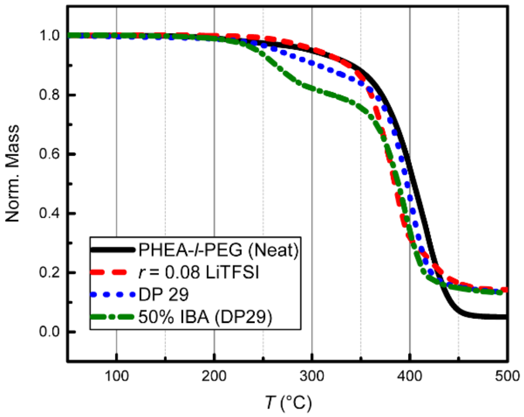

Figure S2: TGA of 4 SPEs – SPE-1 (neat), SPE-1 (*r* = 0.08), SPE-3 (*r* = 0.08), SPE-4 (50% IBA, r = 0.08). Dual-ion samples (SPE-1) are thermally stable to almost 300 °C, while single-ion samples (SPE-3, SPE-4) are thermally stable up to 200 °C. Heating rate 10 °C min^{-1}.

Limiting current fraction and transference numbers

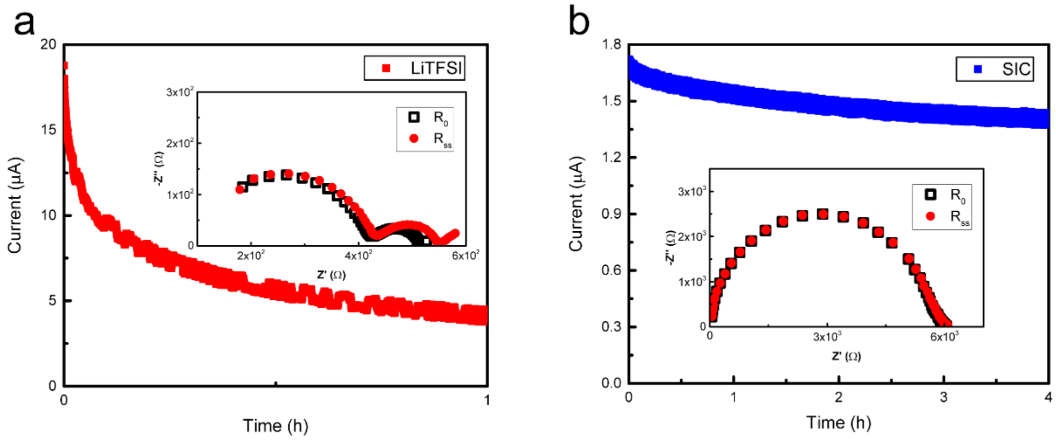

Figure S3: Polarization and EIS for limiting current fraction and transference number of a) SPE-1 and b) SPE-3. The test was performed in a symmetrical Li/SPE/Li cell (r = 0.08) at 70 °C, with a voltage of 10 mV.

Conversion of the Polymerization

Extraction: The incorporation of 2-HEA and PEG into solid PHEA-*l*-PEG (SPE-1, neat) networks was confirmed by extraction using various good solvents. To this end, SPE-1 was immersed in either THF, a 1:1 H_2O/DMF mixture, or MEK for about 24 h exchanging the solvent at least twice. The mass reduction, as determined from weighing the sample before and after extraction following vacuum drying at room temperature for at least 24 h, was found to be less than 13 %. This is significantly less than the nominal PHEA weight fraction of approx. 50 wt% in the (neat) SPE-1 (see Scheme 1), which confirms successful incorporation of the precursor materials into the polymer network.

For all SPEs, the conversion of the polymerization was additionally confirmed based on ATR-FTIR of all SPEs as well as their precursors.

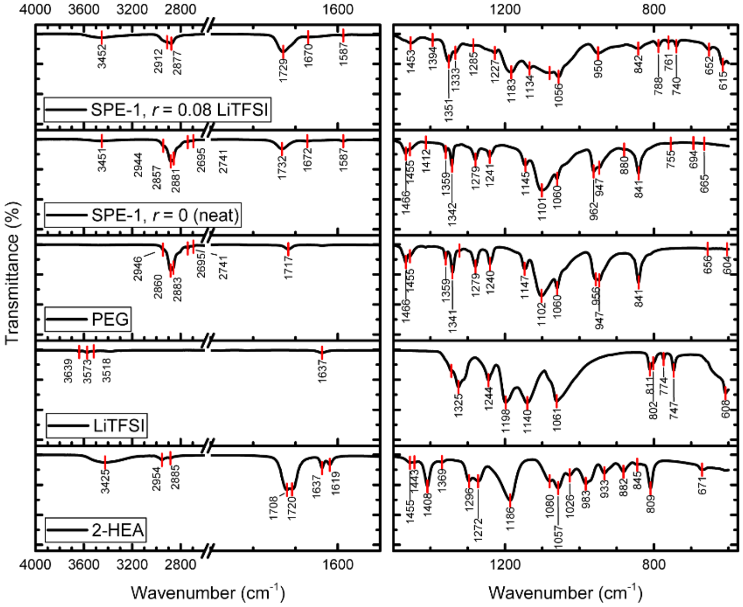

Figure S4: ATR-FTIR of 2-HEA, LiTFSI, PEG, neat SPE-1 ($r = 0$), and LiTFSI-doped SPE-1 ($r = 0.08$).

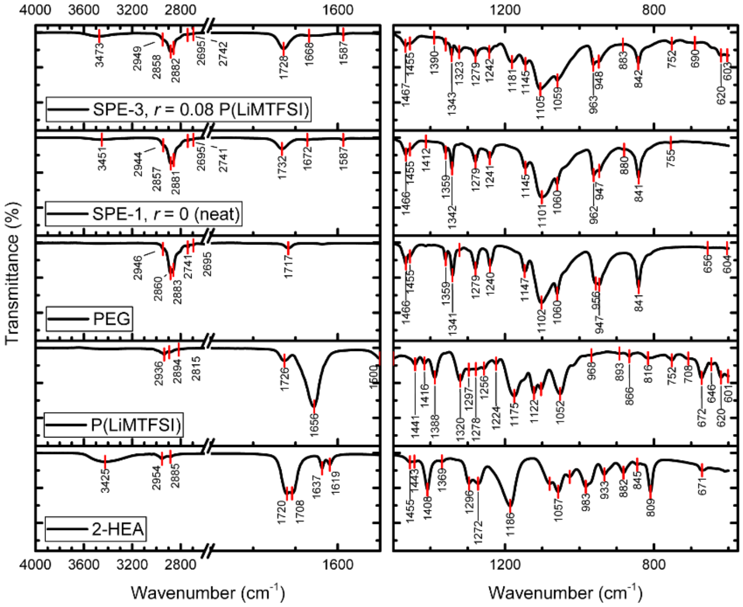

Figure S5: ATR-FTIR of 2-HEA, P(LiMTFSI), PEG, neat SPE-1 ($r = 0$), and SPE-3 containing P(LiMTFSI) ($r = 0.08$).

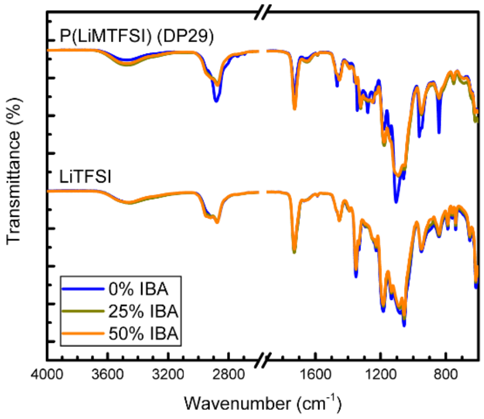

Figure S6: ATR-FTIR of SPE-2 (containing LiTFSI and 25% or 50% IBA; bottom) and SPE-4 (containing P(LiMTSFI and 25% or 50% IBA; top) compared to that of SPE-1 (0% IBA) at a constant Li-ion concentration of $r = 0.08$.

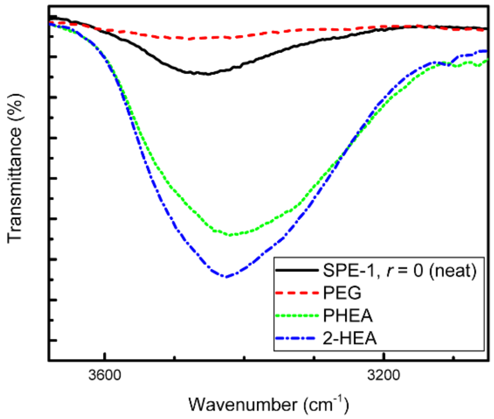

Figure S7: ATR-FTIR of 2-HEA, PHEA, PEG, and neat SPE-1 ($r = 0$) in the hydroxyl group stretching region.

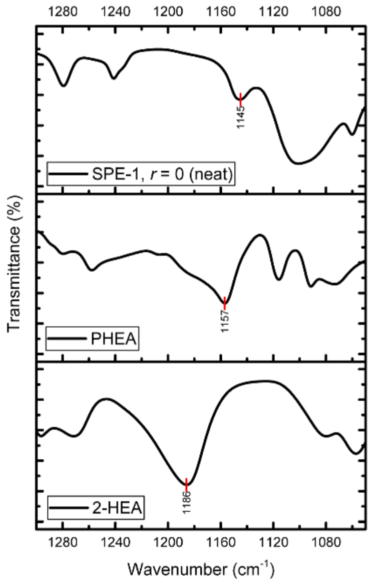

Figure S8: ATR-FTIR of 2-HEA, PHEA, and neat SPE-1 ($r = 0$) in the stretching region of the carbon-oxygen bond (indexed) of the 2-HEA monomer.

Monomer conversion: The absorption band at 1637 cm^{-1} in the spectra of the 2-HEA monomer, which corresponds to carbon double bond stretching, is absent in all SPEs irrespective of Li-ion concentration (Figures S4 and S5) confirming complete monomer conversion. [1-2] The same behavior was observed when comparing the spectra of IBA (not shown) and IBA-containing SPEs (SPE-2 and SPE-4), which were generally found to be very similar to those of SPEs containing only 2-HEA (Figure S6). Incorporation of 2-HEA monomer was confirmed by the broad hydroxyl group stretching band between 3100 cm^{-1} and 3600 cm^{-1},[1-2] which was found in the spectra of 2-HEA and all SPEs but not in those of the other precursor materials (Figures S4, S5, S6 and S7). The relatively strong peak at 1720 cm^{-1} in the spectra of 2-HEA, which corresponds to stretching of the carbonyl group,[2] was found at slightly shifted positions in the spectra of the SPEs and PEG (Figures S4 and S5). While both peaks are less intense than in the case of 2-HEA, the intensity of the former is higher than that of the latter, which further corroborates incorporation of 2-HEA into the SPEs. The relatively strong peak at 1186 cm^{-1} in the 2-HEA spectra, which corresponds to stretching of the carbon-oxygen single bond in ester groups,[2] appears to shift significantly upon polymerization into both linear PHEA as well as cross-linked PHEA-*l*-PEG networks (Figure S8).

PEG incorporation: The characteristic methylene bands of PEG at ~2900 cm^{-1} (stretching),[3-5] ~1460 cm^{-1} (bending),[3, 5] and ~840 cm^{-1} (rocking)[3, 5] can be observed in all SPEs (Figure S4, S5 and S6) confirming the successful incorporation of PEG into the networks. Similarly, PEG absorptions in the range of 1050 cm^{-1} to 1150 cm^{-1}, which correspond to stretching of ether groups,[4] were also observed in the SPEs. While these characteristic absorptions of PEG were found to remain unchanged in the neat SPE-1 ($r = 0$) as well as in P(LiMTFSI)-containing SPE-3 and SPE-4, they slightly shifted upon LiTFSI addition (SPE-1, $r = 0.08$) (Figures S4, S5, and S6). Such salt-induced conformational changes are also observed in PEG-LiTFSI homopolymer electrolytes,[3, 5] which confirms the complexation of PEG and LiTFSI in SPE-1 and SPE-2.

LiTFSI/P(LiTMFSI) incorporation: Confirmation of salt complexation and incorporation of P(LiMTSFI), respectively, is seen in the fingerprint region of the IR spectra (Figure S4, S5, and S6). Several bands resulting from $TFSI^-$ anion vibrations are observed in both, the spectra of LiTFSI and P(LiMTSFI) and the corresponding SPEs, for example, between 1350 cm^{-1} – 1300 cm^{-1} (SO_2 asymmetric stretching),[3, 6] at ~1190 cm^{-1} (CF_3 asymmetric stretching),[3] and ~740 cm^{-1} (CF_3 symmetric deformation).[3, 6] This is consistent with IR spectra of PEG-LiTFSI complexes,[3] and other P(LiMTFSI)-containing solid polymer electrolytes.[7]

References

1. Bruns, N.; Scherble, J.; Hartmann, L.; Thomann, R.; Iván, B.; Mülhaupt, R.; Tiller, J. C., Nanophase Separated Amphiphilic Conetwork Coatings and Membranes. *Macromolecules* **2005,** *38* (6), 2431-2438.

2. Vargün, E.; Usanmaz, A., Polymerization of 2-hydroxyethyl acrylate in bulk and solution by chemical initiator and by ATRP method. *J Polym Sci Part Polym Chem* **2005,** *43* (17), 3957-3965.

3. Rey, I.; Lassègues, J. C.; Grondin, J.; Servant, L., Infrared and Raman study of the PEO-LiTFSI polymer electrolyte. *Electrochim Acta* **1998,** *43* (10-11), 1505-1510.

4. Mansur, H. S.; Oréfice, R. L.; Mansur, A. A. P., Characterization of poly(vinyl alcohol)/poly(ethylene glycol) hydrogels and PVA-derived hybrids by small-angle X-ray scattering and FTIR spectroscopy. *Polymer* **2004,** *45* (21), 7193-7202.

5. Wen, S. J.; Richardson, T. J.; Ghantous, D. I.; Striebel, K. A.; Ross, P. N.; Cairns, E. J., FTIR characterization of PEO + LiN(CF3SO2)2 electrolytes. *J Electroanal Chem* **1996,** *408* (1-2), 113-118.

6. Wang, Z.; Gao, W.; Huang, X.; Mo, Y.; Chen, L., Spectroscopic studies on interactions and microstructures in propylene carbonate?LiTFSI electrolytes. *J Raman Spectrosc* **2001,** *32* (11), 900-905.

7. Porcarelli, L.; Aboudzadeh, M. A.; Rubatat, L.; Nair, J. R.; Shaplov, A. S.; Gerbaldi, C.; Mecerreyes, D., Single-ion triblock copolymer electrolytes based on poly(ethylene oxide) and methacrylic sulfonamide blocks for lithium metal batteries. *J Power Sources* **2017,** *364* (Chem. Mat. 22 2010), 191-199.
